# Preoperative Routine Laboratory Markers for Predicting Postoperative Recurrence and Death in Patients with Breast Cancer

**DOI:** 10.3390/jcm10122610

**Published:** 2021-06-13

**Authors:** Young-Chul Yoo, Seho Park, Hyun-Joo Kim, Hyun-Eom Jung, Ji-Young Kim, Myoung-Hwa Kim

**Affiliations:** 1 Department of Anesthesiology and Pain Medicine, Anesthesia and Pain Research Institute, Yonsei University College of Medicine, 50-1 Yonsei-ro, Seodaemun-gu, Seoul 03722, Korea; seaoyster@yuhs.ac (Y.-C.Y.); JJOLLONG@yuhs.ac (H.-J.K.); 2 Devision of Breast Cancer, Department of General Surgery, Anesthesia and Pain Research Institute, Yonsei University College of Medicine, 50-1 Yonsei-ro, Seodaemun-gu, Seoul 03722, Korea; PSH1025@yuhs.ac; 3 Department of Anesthesiology and Pain Medicine, Yonsei University College of Medicine, Gangnam Severance Hospital, 211 Eonju-ro, Gangnam-gu, Seoul 06273, Korea; EOM4531@yuhs.ac (H.-E.J.); AMYJYKIM@yuhs.ac (J.-Y.K.); 4 Department of Anesthesiology and Pain Medicine, Anesthesia and Pain Research Institute, Yonsei University College of Medicine, Gangnam Severance Hospital, 211 Eonju-ro, Gangnam-gu, Seoul 06273, Korea

**Keywords:** breast cancer, cancer antigen 15-3, death, recurrence, red cell distribution width

## Abstract

Simple, convenient, and reliable preoperative prognostic indicators are needed to estimate the future risk of recurrences and guide the treatment decisions associated with breast cancer. We evaluated preoperative hematological markers related to recurrence and mortality and investigated independent risk factors for recurrence and mortality in patients after breast cancer surgery. We reviewed electronic medical records of patients with invasive breast cancer diagnosed at our tertiary institution between November 2005 and December 2010 and followed them until 2015. We compared two groups of patients classified according to recurrence or death and identified risk factors for postoperative outcomes. Data from 1783 patients were analyzed ultimately. Cancer antigen (CA) 15-3 and red cell distribution width (RDW) had the highest area under the curve values among several preoperative hematological markers for disease-free survival and overall survival (0.590 and 0.637, respectively). Patients with both preoperative CA 15-3 levels over 11.4 and RDW over 13.5 had a 1.7-fold higher risk of recurrence (hazard ratio (HR): 1.655; 95% confidence interval (CI): 1.154–2.374; *p* = 0.007) and mortality (HR: 1.723; 95% CI: 1.098–2.704; *p* = 0.019). In conclusion, relatively high preoperative RDW (>13.5) and CA 15-3 levels (>11.4) had the highest predictive power for mortality and recurrence, respectively. When RDW and CA 15-3 exceeded the cut-off value, the risk of recurrence and death also increased approximately 1.7 times.

## 1. Introduction

Breast cancer accounts for approximately 1 in 3 cancers [1,2] and is now regarded as the most common cancer globally; its incidence increased from 1.7 million in 2005 to 2.4 million cases in 2015 [3]. Above all, breast cancer is currently the main cause of cancer-related mortality in women [3]. Mercifully, overall breast cancer-related death rates decreased by 36% from 1989 to 2012 due to enhancements in early detection and systemic treatments [4,5]. However, nearly 20% of breast cancer patients are diagnosed at advanced stages and experience recurrence or distant metastasis within 5 years [6]. Both ipsilateral breast tumor recurrence and other locoregional recurrence are associated with significantly increased risk of distant disease and death [7,8]. Hence, for patients with breast cancer, outcome assessment is essential because it certainly impacts treatment decisions and prognosis. 

The traditional prognostic factors for breast cancer are age, axillary lymph node status, tumor size, histological features (especially histological grade and lympho-vascular invasion), and molecular subtype including hormone receptor status and human epidermal growth factor receptor 2 expression [9,10,11,12]. In addition to demographic and basic clinical information, an increasing number of novel prognostic markers including hematological markers and perioperative anesthetic information have been explored and identified [13,14]. Recently, incorporation of various genetic and molecular biomarkers was attempted to develop new prognostic models for breast cancer [15]. However, most markers are not applied in clinical practice routinely, and their applicability may be limited due to high cost and the need for specialized expertise and equipment. A quick diagnosis of breast cancer is based on identification of inexpensive biomarkers [16]. Recently, accumulating evidence demonstrated that cancer-related systemic inflammation plays a significant role in the development and progression of several neoplastic diseases including breast cancer [17]. Therefore, there is a need to establish low-cost and simple prognostic indicators for breast cancer using routine hematological markers in a complete blood count. 

We aimed to investigate preoperative hematological predictors for disease-free survival (DFS) and overall survival (OS) and identify independent risk factors for recurrence and mortality after breast cancer surgery.

## 2. Materials and Methods

### 2.1. Study Design

Our study was approved by the Hospital Research Ethics Committee and Institutional Review Board of Severance Hospital, Yonsei University Health System (approval number 3-2020-0134). The informed consent was waived in view of the retrospective nature of this study. We collected clinical data from the electronic medical records of consecutive patients with curative breast cancer surgery between November 2005 and December 2010. The follow-up documents were reviewed until December 2015. Patients who underwent multiple procedures, had incomplete information for anesthesia or surgery, or lacked preoperative hematological data were excluded. 

### 2.2. Data Collection

Data related to the following patients’ demographic information were collected and reviewed: age; body mass index (BMI); presence of comorbidities; values of preoperative routine hematologic markers within 30 days before surgery, i.e., hemoglobin, hematocrit, red blood cell (RBC) count, RBC distribution width (RDW), platelet distribution width (PDW), white blood cell (WBC) count, neutrophil, lymphocyte, neutrophil-to-lymphocyte ratio (NLR), platelet, mean platelet volume (MPV), prothrombin time (PT), alkaline phosphatase (ALP), carcinoembryonic antigen (CEA), and cancer antigen (CA) 15-3; and administered anesthetics and analgesics. Surgical and histopathological information was also collected, including the conducted surgical procedures and the operating time, tumor receptor expression status including estrogen receptor (ER), progesterone receptor (PR), and human epidermal growth factor receptor (HER) 2, tumor-node-metastasis (TNM) stage, type of tumor, histological type, and any chemotherapy or radiotherapy performed. We used the American Joint Committee on Cancer 7th edition criteria to identify the TNM stage [18]. Postoperative assessments included medical history, physical examination, and laboratory and imaging tests to detect any evidence of recurrence. Local metastasis was deemed to have occurred if there was tumor presence in the ipsilateral breast, regional lymph nodes, and/or chest wall. Any recurrence at a distant site, including the contralateral axillary or supraclavicular lymph nodes, was defined as distant metastasis. Recurrence-free survival (RFS) was calculated from the date of surgery to the date when locoregional or distant metastasis was first detected. OS was defined as the interval between the date of the first curative surgery and the date of the last follow-up or death.

### 2.3. Statistical Analysis

Patients were categorized into two groups depending on whether they had recurrence (or died) or not. These two groups were compared using the independent two-sample *t*-test and *χ*^2^ test (Fisher’s exact test) for continuous variables and categorical variables, respectively. 

The Contal and O’Quigley method was used to designate the point where the Kaplan–Meier survival curves of the two groups differed the most with the log-rank test and survival optimal cut-off values obtained. Sensitivity and specificity were calculated for each point using Youden Index cut-off values in the receiver operating characteristic (ROC) curve and area under curve (AUC) where the sum that reached the maximum was assessed. Additionally, predictive power was calculated using Harrell’s C-index with the bootstrapping method. The closer the C-index value was to 1.0, the greater the predictive power was. All potential confounders (chosen based on their clinical significance as reported in the literature) associated with recurrence and mortality after breast cancer surgery, which were age, BMI, co-morbidity, anesthetic agent, analgesics, transfusion, steroid, surgical data, surgical procedure, TNM stage, three receptor status, radiotherapy, neoadjuvant chemotherapy, adjuvant therapy, and histological grade, were analyzed using univariate and multivariate Cox regression methods. First, we performed univariate analysis to identify potential risk factors for postoperative recurrence and mortality; variables with *p*-values < 0.05 were further subjected to multivariate analysis, following which hazard ratio (HR) and the associated 95% confidence interval (CI) were calculated. Multivariate Cox regression processes were additively adjusted for several variables that could be confounding factors. For stepwise multivariate Cox regression analysis, variables including surgical procedure, TNM stage, estrogen receptor, and histological grade were adjusted.

We also conducted propensity score matching analysis for sensitivity test to assess the robustness of our findings regarding the relationship between variables and recurrence (or mortality). After factors including age, BMI, and comorbidities were matched; the greedy heuristic algorithm was used to identify the optimally matched groups without dropouts. This aided in excluding cases with differences that exceeded twice the standard deviation (SD) while matching similar propensity scores. As a result, a 1 to 5 matching score was chosen because it carried the strongest statistical power.

All statistical analyses were conducted with SAS, version 9.4 (SAS Institute Inc., Cary, NC, USA), except for Kaplan–Meier curves, which were constructed using the R statistics (R studio, Boston, MA, USA), version 3.6.2 (www.r-project.org, accessed on 13 June 2020).

## 3. Results

### 3.1. Subjects

**Figure 1:**
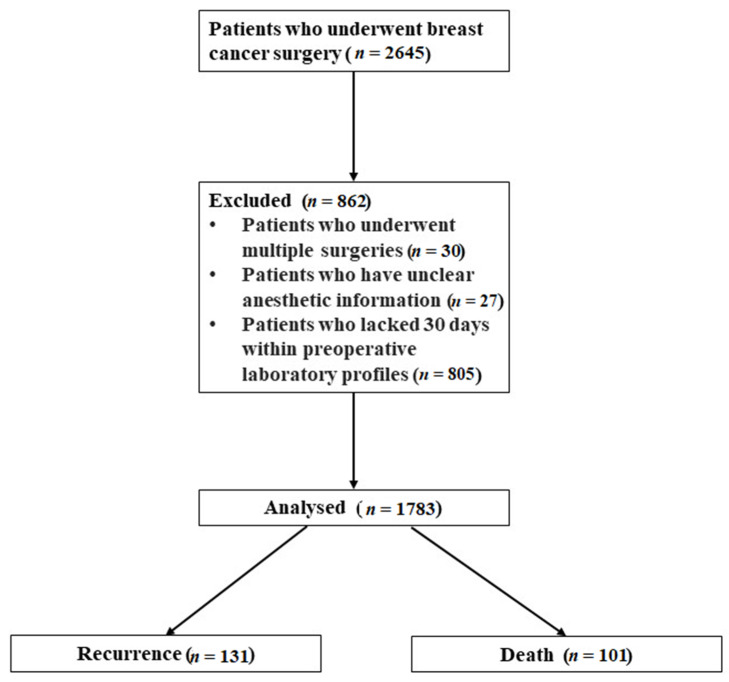
CONSORT Flow Diagram.

We collected the data of 2645 patients who underwent surgery after breast cancer during the study period. Thirty patients who underwent multiple surgeries, 27 with unclear anesthetic methods, and 805 who lacked laboratory profiles within the 30 days before the surgery were excluded. Finally, 1783 patients were analyzed. The mean (SD) follow-up period for our study population was 68.7 (17.2) months. During the study period, the postoperative recurrence rate was 7.5% and the all-cause mortality rate was 5.7% (Figure 1).

### 3.2. Comparison of Patients’ Characteristics

A comparison of the characteristics of patients who had recurrence (*n* = 134) with those who did not have recurrence (*n* = 1649) is shown in Table 1. There were no significant differences regarding the demographic data and anesthetic information. Values of hemoglobin and CA 15-3 and surgical information including conducted procedure, TNM staging, estrogen and progesterone receptor status, histological grade, and status of neo/adjuvant chemotherapy were significantly different between the two groups even after 1 to 5 propensity score matching. 

A comparison of the characteristics of patients who died (*n* = 101) and those who survived (*n* = 1682) is shown in Table 2. The groups were comparable in terms of demographic data and anesthetic information. Hemoglobin, hematocrit, RBC count, RDW, MPV, ALP, and CA 15-3 were significantly different between the two groups. Moreover, surgical factors including the procedure performed, TNM stage, estrogen and progesterone receptor status, histological grade, and status of neo/adjuvant chemotherapy were also significantly different between the two groups even after 1 to 5 propensity score matching.

### 3.3. Predictors for Postoperative Recurrence and Mortality after Breast Cancer Surgery

**Figure 2:**
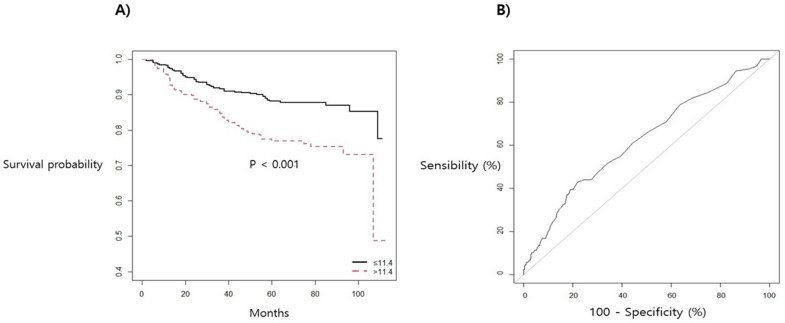
(**A**) Kaplan–Meier survival curve and (**B**) ROC curve for postoperative recurrence in association with CA 15-3 in patients with breast cancer surgery.

**Figure 3:**
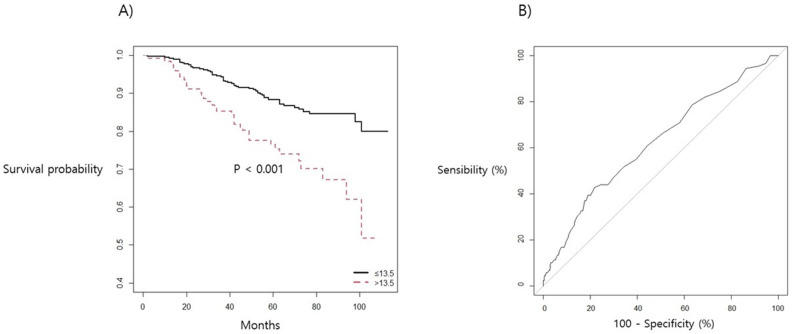
(**A**) Kaplan–Meier survival curve and (**B**) ROC curve for postoperative mortality in association with RDW in patients with breast cancer surgery.

Figure 2 shows the Kaplan–Meier curve with a survival optimal cut-off value and ROC curve of CA 15-3, which was the biomarker with the highest predictive power for postoperative recurrence. When the survival optimal cut-off value of CA15-3 was 11.4 (*p* value < 0.001), the Harrell’s C-index (AUC) was 0.590. RDW showed the highest predictive power regarding postoperative mortality, and the survival optimal cut-off value was 13.5 (*p* value < 0.001). The Harrell’s C-index of RDW was 0.637 (Figure 3). 

### 3.4. Independent Risk Factors for Postoperative Recurrence and Mortality in Breast Cancer Surgery

Table 3 lists the factors that affected postoperative breast cancer recurrence as revealed by competing risk analysis. Multivariate Cox regression with 1 to 5 propensity score matching showed that perioperative CA 15-3 level > 11.4 (HR, 1.665; 95% CI, 1.154–2.374; *p* = 0.007), mastectomy (HR, 2.169; 95% CI, 1.419–3.314; *p* < 0.001) compared with breast-conserving surgery, TNM stage 3 (HR, 3.481; 95% CI, 2.136–5.672; *p* < 0.001) compared with TNM stage 1, neoadjuvant chemotherapy (HR, 1.957; 95% CI, 1.127–3.397; *p* = 0.045), and moderate (HR, 2.071; 95% CI, 1.139–3.766; *p* = 0.017) or poor histological grade (HR, 3.878; 95% CI, 2.044–7.357; *p* < 0.001) compared with good histological grade increased the risks for cancer recurrence. 

The factors associated with postoperative mortality after breast cancer surgery are presented in Table 4. Multivariate Cox regression analysis with 1 to 5 propensity score matching showed that RDW > 13.5 (HR, 1.723; 95% CI, 1.098–2.704; *p* = 0.019), NLR > 2.82 (HR, 1.771; 95% CI, 1.108–2.832; *p* = 0.017), ALP > 76 (HR, 2.257; 95% CI, 1.412–3.607; *p* < 0.001), CEA > 1.57 (HR, 1.553; 95% CI, 1.007–2.395; *p* = 0.047), mastectomy (HR, 2.993; 95% CI, 1.750–5.118; *p* < 0.001) compared with breast-conserving surgery, TNM stage 2 (HR, 2.310; 95% CI, 1.239–4.306; *p* = 0.009) and 3 (HR, 6.563; 95% CI, 3.482–12.370; *p* < 0.001) compared with TNM stage 1, neoadjuvant chemotherapy (HR, 1.442; 95% CI, 1.048–3.409; *p* = 0.035), and moderate (HR, 3.253; 95% CI, 1.412–7.491; *p* = 0.006) and poor histological grade (HR, 6.455; 95% CI, 2.757–15.113; *p* < 0.001) compared with good histological grade remained significant factors for an increased risk of postoperative mortality.

## 4. Discussion

In our study, factors including preoperative routine hematologic markers affecting the prognosis after breast cancer surgery were investigated through follow-up for over 5 years. Compared with previous studies related to the outcomes of oncologic patients using blood tests, our study calculated the survival optimal cut-off value and HR of hematological markers as well as the predictive power of long-term recurrence and mortality after breast surgery using a large sample size. Preoperative relatively high RDW (>13.5) had the highest predictive power for postoperative mortality, and preoperative relatively high CA 15-3 levels (>11.4) had the highest predictive power for recurrence. When these two factors exceeded the cut-off value the risk of recurrence and death also increased approximately 1.7 times. 

Even though many novel prognostic markers have been explored and identified, the main problem in most of these studies is that the biomarkers depend on complex molecular or genetic tests [12,19,20,21,22]. In our study, considering that CA 15-3 and RDW are easily available and inexpensive markers and parameters that are routinely assessed in blood examination, they could be used as potential novel accurate and reproducible indices to identify breast cancer patients with poorer outcomes. 

Complete blood count is essentially used to assess anemia-related diseases, which usually have increased risk for surgical mortality and can often be treated before surgery [23,24]. We have used RDW, which describes the size variability of circulating red blood cells, for the workup of anemia and it has recently been suggested as an inflammatory biomarker [25]. Another mechanism for the association between chronic inflammation and elevated RDW could be increased red blood cell fragility in inflammatory states secondary to the rise of lipid oxidation [26]. Additionally, oxidation of hemoglobin from exposure to free radicals [27] signals erythrocyte removal and increases RDW. Other explanations for the relationship include the increased release and binding of free histones to erythrocytes, which increases their fragility [28]. Similar to inflammation, the status of oxidative stress may reduce RBC survival and lead to elevated RDW [29].

Inflammation in the tumor microenvironment promotes tumor growth, invasion, angiogenesis, and, ultimately, metastasis [30,31,32,33]. It is now extensively recognized that smoldering inflammation plays an important role in the initiation and progression of cancer [31,33]. In the same manner, Seretis et al. [23] reported RDW to be a useful biomarker in distinguishing benign and malignant breast tumors. RDW has also recently been reported as a potential prognostic factor in breast cancer [34,35]. Furthermore, RDW is considered a biomarker that influences the increased mortality rate in non-cardiac surgery because anemia, malnutrition, and oxidative stress may be indicated by high RDW [36]. However, our RDW cut-off value (13.5) was relatively low compared to those reported in other studies (13.75, 13.82, and 13.7). Accordingly, we should pay more attention and monitor patients that have higher preoperational RDW and try to optimize RDW before surgery providing interventions such as nutritional support or anti-inflammatory medications [36]. RDW, which is a potential biomarker of inflammation, might be utilized to risk-stratify cancer surgical patients and monitor the efficacy of risk-modifying strategies. 

Preoperative levels of CA15-3 have an independent association with prognosis in patients with early-stage breast cancer [37]. It has been shown to be extremely sensitive for distant metastases, especially those in the bone and liver [38,39,40]. However, there is currently no clear clinical cut-off for considering CA15-3 abnormal; cut-offs reported in other studies have varied from 22 to 60 U/mL [41,42]. The choice of cut-off to classify CA15-3 could indeed affect the conclusions; a lower cut-off would likely decrease the predictive value, while a higher cut-off may increase the predictive value [43]. In our study, CA 15-3 was associated with the risk of recurrence and had predictive power. The cut-off value was 11.4, and the predicted power was relatively low, with an AUC of 0.59 (negative predictive value 94.8%, positive predictive value 11.4%). Although it was a cut-off value in our population, a slight increase in CA 15-3 before surgery may indicate an increased risk of recurrence. Persistently elevated tumor markers including CA 15-3 are associated with recurrence or treatment resistance, which may be useful in monitoring treatment effects [44]. 

CA 15-3 can be easily and inexpensively tested, but its sensitivity is relatively low [45,46]. The role of CA 15-3 in breast cancer surveillance remains controversial. Moreover, there is no definite guideline on how often to test; this test alone can increase the patient’s anxiety enough. Because there were insufficient data including prospective randomized clinical trials to provide evidence on whether detection and treatment of occult or asymptomatic metastases using CA 15-3 impact on the most significant prognosis, ASCO guidelines for follow-up and management of patients with breast cancer [47] have not recommended the use of CA 15.3 for detecting recurrence routinely. On the contrary, European Group on Tumor Markers has recommended the use of CA15-3 levels for assessing prognosis, early detection of disease progression, and treatment monitoring in breast cancer [44]. It was also reported that the preoperative elevation of CA15-3 was associated with aggressive characteristics and worse overall survival in breast cancer patients without distant metastasis [48,49,50]. Consequently, routine follow-ups of CA 15-3 to check for relapse after treatment of breast cancer have not yet been not recommended by major international guidelines [40,47,51] because of its high frequency of false negative and false positive prediction. 

The Nottingham Prognostic Index (NPI), known for prognostic index, is also widely used for predicting survival in operable primary patients with breast cancer [52,53]. It is based on tumor size, histologic grade, and lymph node status [54] and is simple and easy for clinical use. It shows suboptimal performance in predicting tumor recurrence. The AUCs were 0.66 and 0.63 in the training and test cohorts, respectively [55,56,57,58]. In our findings, RDW had a similar predictive power as that of NPI indicating that information can be easily obtained through blood tests that can approximately predict a patient’s prognosis. Biomarkers alone, however, are insufficient to predict the postoperative prognosis in patients with breast cancer, but correction of RDW before surgery may lower the risk of postoperative death. Moreover, CA 15-3 is highly related to recurrence, and it can act as a useful marker for cancer tracing.

Our study has some limitations, including its retrospective cohort design, which was associated with the selecting bias inclusion of an entire population. Although the propensity matching score carefully balanced age, sex, and a large host of preoperative comorbidities that might affect the relationship between RDW and mortality, there still might be unbalanced preoperative comorbidities or medications explaining the relationship. These could include differences in the cofactors of hemoglobin production or ischemic diseases. Therefore, it needs future investigation in a less heterogeneous cohort. Another limitation is that we did not investigate precise mechanisms including molecular or genetic explanations regarding how CA 15-3 and RDW act on the oncological outcomes, especially on breast cancer. In addition, the association scores of multiple variables should be more clearly identified because CA 15-3 was one of the several significant variables included in the multivariate Cox regression analysis including propensity matching. Further prospective studies are needed to determine the role of hematological markers for predictors. 

The calculated RDW cut-off value related to the risk of death was 13.5, which was lower than that reported previously. This shows that even a slight increase in RDW in breast cancer patients may have a correlation with postoperative mortality. There may be several reasons for the increase in RDW; therefore, efforts to normalize RDW before surgery are necessary at least in patients with breast cancer. CA15-3 was associated solely with the risk of breast cancer recurrence in our study. Although the AUC was relatively low for predicting recurrence, monitoring of CA 15-3 levels would be somewhat helpful in the follow-ups for breast cancer recurrence or metastasis. 

## Figures and Tables

**Table 1:** Patient characteristics based on postoperative recurrence.

	Before 1 to 5 Propensity Score Matching			After 1 to 5 Propensity Score Matching		
Parameters	Non-Recurrence (*n* = 1649)	Recurrence (*n* = 134)	*p*-Value	Non-Recurrence (*n* = 645)	Recurrence (*n* = 129)	*p*-Value
Demographic data						
Age (yr)	50.1 ± 9.9	49.7 ± 11.2	0.683	48.7 ± 9.49	49.69 ± 10.88	0.333
Body mass index (kg/m^2^)	23.3 ± 3.2	23.0 ± 3.0	0.244	23.0 ± 3.1	23.1 ± 3.0	0.851
Comorbidities						
Hypertension	321 (19.5)	30 (22.4)	0.413	114 (17.7)	27 (20.9)	0.382
Diabetes mellitus	110 (6.7)	13 (9.7)	0.183	37 (5.7)	12 (9.3)	0.129
Cardiac	43 (2.6)	4 (3.0)	0.777	18 (2.8)	4 (3.1)	0.775
Pulmonary	27 (1.6)	4 (3.0)	0.287	10 (1.5)	0 (0.0)	0.384
Endocrine	79 (4.8)	7 (5.2)	0.822	26 (4.0)	7 (5.4)	0.474
Renal	10 (0.6)	0 (0.0)	>0.999	0 (0.0)	0 (0.0)	>0.999
Liver	8 (0.5)	1 (0.8)	0.506	3 (0.5)	1 (0.8)	0.519
Neurological	24 (1.5)	2 (1.5)	>0.999	9 (1.4)	1 (0.8)	>0.999
Others	10 (0.6)	1 (0.8)	0.578	3 (0.5)	1 (0.8)	0.519
Hematologic markers						
Hemoglobin (g/dl)	12.9 ± 1.2	12.6 ± 1.4	0.032	12.8 ± 1.2	12.6 ± 1.5	0.098
Hematocrit (%)	39.0 ± 3.3	38.2 ± 4.2	0.023	38.9 ± 3.2	38.1 ± 4.2	0.070
RBC count (10^6^/µL)	4.33 ± 0.38	4.23 ± 0.49	0.018	4.33 ± 0.37	4.23 ± 0.50	0.027
RDW (%)	13.16 ± 1.35	13.55 ± 1.80	0.014	13.20 ± 1.37	13.53 ± 1.80	0.051
PDW (fL)	11.12 ± 1.45	10.97 ± 1.50	0.265	11.09 ± 1.44	10.97 ± 1.49	0.358
WBC count (10^3^/µL)	6.01 ± 1.74	6.00 ± 1.83	0.983	6.12 ± 1.88	6.01 ± 1.85	0.563
Neutrophil (%)	3.67 ± 1.48	3.75 ± 1.50	0.550	3.75 ± 1.57	3.76 ± 1.52	0.982
Lymphocyte (%)	1.86 ± 0.60	1.76 ± 0.65	0.067	1.88 ± 0.61	1.78 ± 0.66	0.082
NLR	2.16 ± 1.20	2.41 ± 1.49	0.065	2.18 ± 1.17	2.40 ± 1.51	0.109
Platelet (10^3^/µL)	258.17 ± 60.17	252.90 ± 72.14	0.413	262.55 ± 63.35	252.12 ± 72.70	0.131
MPV (fL)	9.77 ± 0.86	9.57 ± 1.06	0.039	9.75 ± 0.86	9.57 ± 1.06	0.079
PT (sec)	11.00 ± 0.76	11.05 ± 0.74	0.540	11.04 ± 0.83	11.05 ± 0.74	0.853
ALP (IU/L)	59.97 ± 20.02	63.52 ± 20.29	0.049	59.14 ± 19.26	63.22 ± 19.89	0.030
CEA (ng/mL)	1.74 ± 3.04	1.91 ± 2.88	0.539	1.62 ± 1.77	1.92 ± 2.93	0.253
CA 15-3 (U/mL)	11.97 ± 6.55	13.78 ± 7.66	0.009	11.95 ± 7.07	13.75 ± 7.68	0.010
Anesthetic information						
Anesthetic agent			0.554			0.480
Sevoflurane	1017 (61.7)	76 (56.7)		399 (61.9)	75 (58.1)	
TIVA	20 (1.2)	3 (2.2)		7 (1.1)	2 (1.6)	
Desflurane	442 (26.8)	38 (28.4)		190 (29.5)	36 (27.9)	
Isoflurane	151 (9.2)	14 (10.5)		40 (6.20)	13 (10.08)	
Enflurane	19 (1.2)	3 (2.2)		9 (1.40)	3 (2.33)	
Analgesic agents						
NSAIDs	1486 (90.1)	125(93.3)	0.230	581 (90.1)	120 (93.0)	0.296
Opioids	1023 (62.0)	87(64.9)	0.508	387 (60.0)	86 (66.7)	0.157
Tramadol	625 (37.9)	43(32.1)	0.182	255 (39.5)	43 (33.3)	0.187
Dexamethasone	138 (8.4)	14(10.5)	0.407	51 (7.9)	13 (10.1)	0.414
Surgical information						
Procedure			<0.001			<0.001
BCS	851 (51.6)	33 (24.6)		329 (51.0)	32 (24.8)	
Mastectomy	798 (48.4)	101 (75.4)		316 (49.0)	97 (75.2)	
Surgical time	207.8 ± 134.5	221.3 ± 121.9	0.263	204.6 ± 125.3	223.3 ± 123.4	0.120
TNM stage			<0.001			<0.001
1	849 (51.5)	31 (23.1)		310 (48.1)	29 (22.5)	
2	617 (37.4)	49 (36.6)		258 (40.0)	47 (36.4)	
3	183 (11.1)	54 (40.3)		77 (11.9)	53 (41.1)	
Receptor status						
Estrogen	1185 (71.86)	78 (58.2)	<0.001	469 (72.7)	75 (58.1)	<0.001
Progesterone	1081 (65.55)	72 (53.7)	0.006	429 (66.5)	71 (55.0)	0.013
HER2	455 (27.59)	31 (23.1)	0.265	156 (24.2)	31 (24.0)	0.970
Histological grade			<0.001			<0.001
Well	369 (22.4)	14 (10.5)		152 (23.6)	14 (10.9)	
Moderate	740 (44.9)	59 (44.0)		289 (44.8)	55 (42.6)	
Poorly	369 (22.4)	57 (42.5)		142 (22.0)	56 (43.4)	
Other	171 (10.4)	4 (3.0)		62 (9.6)	4 (3.1)	
Neoadjuvant chemotherapy	64 (3.9)	19 (14.2)	<0.001	25 (3.9)	18 (14.0)	<0.001
Adjuvant chemotherapy	937 (56.8)	98 (73.1)	<0.001	392 (60.8)	96 (74.4)	0.004
Radiotherapy	1062 (64.4)	83 (61.9)	0.568	429 (66.5)	80 (62.0)	0.326

Data are presented as mean (standard deviation) or number (percentage). Propensity score analysis was matched by factors including age, body mass index, and comorbidities.

**Table 2:** Patient characteristics based on postoperative mortality.

	Before 1 to 5 Propensity Score Matching			After 1 to 5 Propensity Score Matching		
Parameters	Non-Death (*n* = 1682)	Death (*n* = 101)	*p*-Value	Non-Death (*n* = 445)	Death (*n* = 89)	*p*-Value
Demographic data						
Age (yr)	49.9 ± 9.8	52.4 ± 12.7	0.054	50.8 ± 9.8	50.9 ± 11.8	0.333
Body mass index (kg/m^2^)	23.3 ± 3.1	22.8 ± 3.1	0.102	22.9 ± 2.9	22.9 ± 3.1	0.851
Comorbidities						
Hypertension	323 (19.2)	28 (27.7)	0.037	110 (24.7)	21 (23.6)	0.382
Diabetes mellitus	110 (6.5)	13 (12.9)	0.015	44 (9.9)	8 (9.0)	0.129
Cardiac	41 (2.4)	6 (5.9)	0.046	4 (0.9)	3 (3.4)	0.775
Pulmonary	25 (1.5)	6 (5.9)	0.007	1 (0.2)	0 (0.0)	0.384
Endocrine	81 (4.8)	5 (5.0)	0.814	17 (3.8)	4 (4.5)	0.765
Renal	9 (0.54)	1 (1.0)	0.443	3 (0.7)	1 (1.1)	0.519
Liver	7 (0.4)	2 (2.0)	0.088	0 (0.0)	0 (0.0)	>0.999
Neurological	24 (1.4)	2 (2.0)	0.656	8 (1.8)	2 (2.3)	0.676
Others	11 (0.7)	0 (0.0)	>0.999	0 (0.0)	0 (0.0)	>0.999
Hematologic markers						
Hemoglobin (g/dl)	12.9 ± 1.2	12.6 ± 1.5	0.069	12.9 ± 1.2	12.5 ± 1.6	0.033
Hematocrit (%)	39.0 ± 3.3	38.1 ± 4.5	0.043	39.0 ± 3.2	37.8 ± 4.6	0.018
RBC count (10^6^/µL)	4.33 ± 0.38	4.19 ± 0.52	0.007	4.34 ± 0.36	4.16 ± 0.53	0.004
RDW (%)	13.15 ± 1.33	13.84 ± 2.05	0.002	13.11 ± 1.22	13.87 ± 2.17	0.002
PDW (fL)	11.12 ± 1.45	10.91 ± 1.53	0.161	11.04 ± 1.44	10.85 ± 1.48	0.283
WBC count (10^3^/µL)	5.98 ± 1.73	6.38 ± 2.01	0.053	6.01 ± 1.57	6.38 ± 2.09	0.122
Neutrophil (%)	3.66 ± 1.47	3.95 ± 1.67	0.060	3.67 ± 1.35	3.98 ± 1.72	0.116
Lymphocyte (%)	1.85 ± 0.59	1.90 ± 0.75	0.559	1.88 ± 0.57	1.88 ± 0.77	0.916
NLR	2.17 ± 1.19	2.44 ± 1.66	0.104	2.14 ± 1.17	2.50 ± 1.73	0.057
Platelet (10^3^/µL)	257.92 ± 59.95	255.32 ± 78.72	0.745	264.28 ± 59.61	257.71 ± 80.41	0.467
MPV (fL)	9.77 ± 0.87	9.42 ± 1.03	0.001	9.73 ± 0.89	9.41 ± 1.03	0.003
PT (sec)	11.01 ± 0.75	11.04 ± 0.78	0.627	10.94 ± 0.78	11.01 ± 0.74	0.407
ALP (IU/L)	59.80 ± 19.81	67.53 ± 22.74	0.002	60.48 ± 18.55	65.62 ± 21.67	0.039
CEA (ng/mL)	1.74 ± 3.09	1.93 ± 1.58	0.284	1.83 ± 4.09	1.78 ± 1.41	0.811
CA 15-3 (U/mL)	11.98 ± 6.53	14.25 ± 8.12	0.007	12.21 ± 6.10	13.98 ± 7.56	0.040
Anesthetic information						
Anesthetic agent			0.107			0.174
Sevoflurane	1037 (61.7)	56 (55.5)		268 (60.2)	50 (56.2)	
TIVA	21 (1.3)	2 (2.0)		4 (0.9)	1 (1.1)	
Desflurane	451 (26.8)	29 (28.7)		128 (28.8)	26 (29.2)	
Isoflurane	155 (9.2)	10 (9.9)		41 (9.2)	8 (9.0)	
Enflurane	18 (1.1)	4 (4.0)		4 (0.9)	4 (4.5)	
Analgesic agents						
NSAIDs	1520 (90.4)	91 (90.1)	0.929	404 (90.8)	83 (93.3)	0.453
Opioids	1044 (62.1)	66 (65.4)	0.510	265 (59.6)	59 (66.3)	0.235
Tramadol	643 (38.2)	25 (24.8)	0.007	179 (40.2)	25 (28.1)	0.032
Dexamethasone	144 (8.6)	8 (7.9)	0.823	34 (7.6)	6 (6.7)	0.769
Surgical information						
Procedure			<0.001			<0.001
BCS	865 (51.4)	19 (18.8)		218 (49.0)	18 (20.2)	
Mastectomy	817 (48.6)	82 (81.2)		227 (51.0)	71 (79.8)	
Surgical time	209.5 ± 135.7	197.7 ± 91.5	0.224	204.6 ± 125.3	223.3 ± 123.4	0.120
TNM stage			<0.001			<0.001
1	862 (51.3)	18 (17.8)		226 (50.8)	14 (15.7)	
2	625 (37.2)	41 (40.6)		166 (37.3)	37 (41.6)	
3	195 (11.6)	42 (41.6)		53 (11.9)	38 (42.7)	
Receptor status						
Estrogen	1215 (72.2)	48 (47.5)	<0.001	336 (75.5)	40 (44.9)	<0.001
Progesterone	1110 (66.0)	43 (42.6)	<0.001	292 (65.6)	38 (42.7)	<0.001
HER2	458 (27.2)	28 (27.7)	0.914	123 (27.6)	25 (28.1)	0.932
Histological grade			<0.001			<0.001
Well	373 (22.2)	10 (9.9)		116 (26.1)	7 (7.8)	
Moderate	760 (45.2)	39 (38.6)		190 (42.7)	33 (37.1)	
Poorly	380 (22.6)	46 (45.5)		92 (20.7)	43 (48.3)	
Other	169 (10.1)	6 (5.9)		47 (10.6)	6 (6.7)	
Neoadjuvantchemotherapy	63 (3.8)	20 (19.8)	<0.001	16 (3.6)	19 (21.4)	<0.001
Adjuvantchemotherapy	965 (57.4)	70 (69.3)	0.019	268 (60.2)	65 (73.0)	0.023
Radiotherapy	1085 (64.5)	60 (59.4)	0.299	273 (61.4)	55 (61.8)	0.937

Data are presented as mean (standard deviation) or number (percentage). Propensity score analysis was matched by factors including age, body mass index, and comorbidities.

**Table 3:** Independent risk factors for patients with breast cancer associated with postoperative recurrence after Cox regression analysis with 1 to 5 propensity score matching.

Parameter		Univariate Analysis		Multivariate Analysis	
		HR (95% CI)	*p*-Value	HR (95% CI)	*p*-Value
Hematologic markers					
RDW (%)	>13.4	1.713 (1.196–2.453)	0.004	1.238 (0.849–1.806)	0.267
ALP (IU/L)	>63	1.475 (1.040–2.093)	0.030	–	
CEA (ng/mL)	>2.73	1.653 (1.060–2.577)	0.027	1.543 (0.988–2.411)	0.056
CA 15-3 (U/mL)	>11.4	2.145 (1.513–3.040)	<0.001	1.655 (1.154–2.374)	0.007
Surgical procedure	BCS	1 (ref)		1 (ref)	
	Mastectomy	2.741 (1.837–4.088)	<0.001	2.169 (1.419–3.314)	<0.001
TNM stage	1	1 (ref)		1 (ref)	
	2	1.781 (1.121–2.831)	0.015	1.173 (0.728–1.891)	0.512
	3	5.863 (3.725–9.226)	<0.001	3.481 (2.136–5.672)	<0.001
Estrogen receptor	Negative	1 (ref)		1 (ref)	
	Positive	0.512 (0.360–0.727)	<0.001	0.671 (0.455–0.990)	0.045
Progesterone receptor	Negative	1 (ref)		1 (ref)	
	Positive	0.598 (0.422–0.846)	0.004	–	
Neoadjuvant chemotherapy		3.090 (1.876–5.089)	<0.001	1.957 (1.127–3.397)	0.018
Adjuvant chemotherapy		1.659 (1.116–2.465)	0.013	–	
Histological grade	Good	1 (ref)		1 (ref)	
	Moderate	2.152 (1.194–3.876)	0.011	2.071 (1.139–3.766)	0.017
	Poor	4.165 (2.312–7.503)	<0.001	3.878 (2.044–7.357)	<0.001
	Other	0.734 (0.242–2.231)	0.586	0.598 (0.194–1.840)	0.370

Propensity score analysis was matched by factors including age, body mass index, and comorbidities.

**Table 4:** Independent risk factors for patients with breast cancer associated with postoperative mortality after Cox regression analysis with 1 to 5 propensity score matching.

Parameter		Univariate Analysis		Multivariate Analysis	
		HR (95% CI)	*p*-Value	HR (95% CI)	*p*-Value
Age	<40	1 (ref)		1 (ref)	
	40–50	0.528 (0.284–0.980)	0.043	–	
	50–60	0.622 (0.330–1.170)	0.141	–	
	60–70	0.701 (0.354–1.389)	0.309	–	
	≥70	1.343 (0.491–3.669)	0.566	–	
Hematologic markers					
Hemoglobin (g/dl)		0.809 (0.697–0.940)	0.006	–	
RDW (%)	>13.5	2.368 (1.546–3.625)	<0.001	1.723 (1.098–2.704)	0.019
WBC count (10^3^/µL)		1.137 (1.009–1.281)	0.035	–	
NLR	>2.82	1.936 (1.238–3.029)	0.004	1.771 (1.108–2.832)	0.017
MPV (fL)	>8.6	0.358 (0.213–0.601)	<0.001	0.469 (0.271–0.811)	0.007
ALP (IU/L)	>76	1.729 (1.104–2.708)	0.017	2.257 (1.412–3.607)	<0.001
CEA (ng/mL)	>1.57	1.590 (1.046–2.417)	0.031	1.553 (1.007–2.395)	0.047
CA 15-3 (U/mL)	>11.5	2.126 (1.396–3.236)	<0.001	1.423 (0.919–2.206)	0.115
Surgical procedure	BCS	1 (ref)		1 (ref)	
	Mastectomy	3.287 (1.959–5.516)	<0.001	2.993 (1.750–5.118)	<0.001
TNM stage	1	1 (ref)		1 (ref)	
	2	3.172 (1.714–5.869)	<0.001	2.310 (1.239–4.306)	0.009
	3	8.676 (4.699–16.019)	<0.001	6.563 (3.482–12.370)	<0.001
Estrogen receptor	Negative	1 (ref)		1 (ref)	
	Positive	0.279 (0.184–0.425)	<0.001	0.350 (0.219–0.561)	<0.001
Progesterone receptor	Negative	1 (ref)		1 (ref)	
	Positive	0.387 (0.254–0.589)	<0.001	–	
Radiotherapy		1.027 (0.669–1.576)	0.904	–	
Neoadjuvant chemotherapy		4.403 (2.649–7.319)	<0.001	1.890 (1.048–3.409)	0.035
Adjuvant chemotherapy		1.533(0.959–2.450)	0.074	–	
Histological grade	Good	1 (ref)		1 (ref)	
	Moderate	2.991 (1.320–6.776)	0.009	3.253 (1.412–7.491)	0.006
	Poor	7.661 (3.430–17.111)	<0.001	6.455 (2.757–15.113)	<0.001
	Other	2.012 (0.676–5.991)	0.209	1.452 (0.480–4.393)	0.509

Propensity score analysis was matched by factors including age, body mass index, and comorbidities.

## Data Availability

The datasets used and/or analyzed during the current study are available from the corresponding author upon reasonable request.

## Abbreviations

RBC	red blood cell
WBC	white blood cell
RDW	red blood cell distribution width
PDW	platelet distribution width
NLR	neutrophil-to-lymphocyte ratio
MPV	mean platelet volume
PT	prothrombin time
ALP	alkaline phosphatase
CEA	carcinoembryonic antigen
CA	cancer antigen
TIVA	total intravenous anesthesia
NSAIDs	non-steroidal anti-inflammatory drugs
BCS	breast-conserving surgery
TNM	tumor-node-metastasis
HER2	human epidermal growth factor receptor 2
CI	confidence interval
HR	hazard ratio
ROC	receiver operating characteristic
